# First records of the genus *Parazygiella* Wunderlich, 2004 (Araneae, Araneidae) from China, with the description of two new species

**DOI:** 10.3897/zookeys.1269.163661

**Published:** 2026-02-13

**Authors:** Xiaoqi Mi, Cheng Wang, Haochun Chen

**Affiliations:** 1 College of Agriculture and Forestry Engineering and Planning, Guizhou Provincial Key Laboratory of Biodiversity Conservation and Utilization in the Fanjing Mountain Region, Tongren University, Tongren 554300, Guizhou, China Guizhou Provincial Key Laboratory of Biodiversity Conservation and Utilization in the Fanjing Mountain Region, Tongren University Tongren China; 2 Central South Academy of Inventory and Planning of National Forestry and Grass Administration, Changsha 410014, Hunan, China Central South Academy of Inventory and Planning of National Forestry and Grass Administration Changsha China

**Keywords:** Arachnida, diagnosis, morphology, new combination, taxonomy, Tibet

## Abstract

The genus *Parazygiella* Wunderlich, 2004 is recorded from China for the first time, with two new species described here from Xizang: *Parazygiella jianxini* Mi & Wang, **sp. nov**. (♂♀) and *P. yanghongae* Mi & Wang, **sp. nov**. (♂♀). Additionally, two species, *P. hiramatsui* (Tanikawa, 2017), **comb. nov**. and *P. nearctica* (Gertsch, 1964), **comb. nov**. are transferred from *Zygiella* F.O. Pickard-Cambridge, 1902.

## Introduction

Zygiellini Simon, 1929 is a small group of araneoid spiders currently classified within the subfamily Phonognathinae Simon, 1894 (WSC 2025) and restricted to the Holarctic region. Historically, it was treated either as a separate subfamily of Araneidae or Tetragnathidae, or even as its own family (WSC 2025), containing only the genus *Zygiella* F.O. Pickard-Cambridge, 1902. The genus was globally revised by Levi (1974). Wunderlich (2004) subsequently split *Zygiella* into four genera—*Leviellus* Wunderlich, 2004, *Parazygiella* Wunderlich, 2004, *Stroemiellus* Wunderlich, 2004, *Zygiella*—and redelimited the family Zygiellidae.

Until recently, only four species of *Zygiella* were known from China (Song et al. 1999), three of which are now placed in *Guizygiella* Zhu, Kim & Song, 1997 (WSC 2025). During our study of araneoid spiders in China, we identified two species from Xizang (Tibet) related to *Zygiella*. A review of the literature indicates that these represent new species, sharing characters with both *Parazygiella* (in having a similar male palp) and *Leviellus* (in possessing similar epigynes with distinct scapes).

The goal of this paper is to describe these two species, which we tentatively place in *Parazygiella*.

## Material and methods

All specimens were collected either by beating shrubs during the daytime or by direct searching at night, and they were preserved in 75% ethanol. The specimens are deposited in the Museum of Tongren University, China (**TRU**). Collection and examination methods mainly follow those of Mi et al. (2023). Photographs of webs or live spiders were taken using a Nikon D6 digital camera with a Nikon Micro-Nikkor 105 mm lens. The distribution map was generated using ArcGIS v. 10.4. Adobe Photoshop CS2.0 was used to edit images and to produce the figures, including the map.

All measurements are given in millimetres. Leg measurements are given as total length (femur, patella + tibia, metatarsus, tarsus). The abbreviations used in the text and figures are as follows: **ALE** anterior lateral eye; **AME** anterior median eye; **C** conductor; **CD** copulatory duct; **CO** copulatory opening; **E** embolus; **FD** fertilization duct; **MA** median apophysis; **MOA** median ocular area; **Pc** paracymbium; **PLE** posterior lateral eye; **PME** posterior median eye; **PP** posterior plate; **R** radix; **Sc** scape; **Sp** spermatheca; **TA** terminal apophysis; **TP** tegular projection.

## Taxonomy

### Family Araneidae Clerck, 1757

**Subfamily Phonognathinae Simon, 1894**

#### Genus Parazygiella

Taxon classificationAnimaliaAraneaeAraneidae

Wunderlich, 2004

683E3AC5-9FFC-5DD0-B9E0-FB1CDA62E13C

Parazygiella Wunderlich, 2004: 936.

##### Type species.

*Zilla montana* C.L. Koch, 1834.

##### Comments.

Gregorič et al. (2015) synonymized this genus with *Zygiella*. Based on the study of trichobothria and shape of copulatory organs, Eskov and Marusik (2024) revalidated *Parazygiella*.

##### Diagnosis.

*Parazygiella* resembles *Zygiella* and *Leviellus* in having an elliptical abdomen with longitudinal dorsal patches extending from anterior to posterior. It can be distinguished from *Zygiella* by the following set of features: paracymbium divided into two lobes with sharp distal edges (Levi 1974: figs 55, 56, 59, 60) vs not divided distally in *Zygiella* (Levi 1974: figs 28–30); palp with a tegular projection (Levi 1974: figs 55, 56, 60) and a terminal apophysis (Levi 1974: figs 55, 56, 60, 61) vs lacking tegular projection and terminal apophysis in *Zygiella* (Levi 1974: figs 28–30); and length of the palp tibia less than half the cymbial length vs at least 2/3 the cymbial length in *Zygiella*. It differs from *Leviellus* by: lacking scape (Levi 1974: figs 51–54) or scape with indistinct border to the epigynal base (Figs 5A–C, 8A–C) vs scape with distinct border to the epigynal base (Levi 1974: fig. 89); and paracymbium divided into two lobes with sharp distal edges (Levi 1974: figs 55, 56, 59, 60) vs not divided distally (Levi 1974: figs 85, 86).

##### Description.

Total length of females 6.2–8.0 and males 4.8–6.8. Carapace pear-shaped, yellow to brown, cephalic region and cervical groove darker than thoracic region, fovea longitudinal (Figs 3A, 3D, 6A, 6D). Endites almost square (Figs 3B, 3E, 6B, 6E). Labium triangular, swollen (Figs 3B, 3E, 6B, 6E). Sternum cordiform, dark brown (Figs 3B, 3E, 6B, 6E). Legs yellow, with dark annuli (Figs 3A, 3D, 6A, 6D). Abdomen elliptical, dorsum with longitudinal patches from anterior to posterior (Figs 3A, 3C, 3D, 3F, 6A, 6C, 6D, 6F). Ventral abdomen yellow to greyish yellow with pale line on each side (Figs 3B, 3E, 6B, 6E).

Male palp with 1 patellar seta (Figs 4A–C, 4E, 7A–C, 7E); tibia at most 1.3× longer than wide (Figs 4A–C, 4E, 7A–C, 7E); paracymbium (Pc) divided into two lobes distally (Figs 4B, 4C, 4E, 7B, 7C, 7E); tegulum with one or two projections (Figs 4A–E, 7A–E); median apophysis (MA) heavily sclerotized, flattened, long axis approximately parallel to cymbium (Figs 4A, 4B, 4E, 7A, 7B, 7E); conductor (C) thick, translucent (Figs 4A, 4B, 4D, 4E, 7A, 7B, 7D, 7E); terminal apophysis (TA) prominent, close to base of embolus (Figs 4A–E, 7A–E); embolus long, flattened, with wide, translucent base, curved over tip of bulb and extending basally nearly half of bulb length (Figs 4A–C, 4E, 7A–C, 7E).

Epigyne heavily sclerotized, without scape except for two new species (Figs 5A–C, 8A–C); copulatory openings situated on dorsal or posterior surface of epigyne (Figs 5B, 8B); copulatory ducts longer than spermathecal diameter (Figs 5D, 8D); spermathecae rounded, about their radius or less apart (Figs 5D, 8D).

##### Note.

The two new species described here differ from the generotype in that the males have a tooth on the endite, and females have a long epigynal scape—characters absent in *P. montana*. They are provisionally assigned to *Parazygiella* because we prioritize palpal morphology over epigynal morphology. Future analysis may justify their transfer to a separate new genus.

##### Composition.

*Parazygiella montana* (C.L. Koch, 1834) (Western Palaearctic), *P. carpenteri* (Archer, 1951) (Western Nearctic), *P. dispar* (Kulczyński, 1885) (eastern Palaearctic and western Nearctic), *P. jianxini* Mi & Wang, sp. nov. (Xizang, China), *P. yanghongae* Mi & Wang, sp. nov. (Xizang, China), *P. hiramatsui* (Tanikawa, 2017), comb. nov. (Amami-oshima Island, Japan) and *P. nearctica* (Gertsch, 1964), comb. nov. (Trans-Nearctic).

##### Distribution.

Holarctic.

#### Parazygiella jianxini

Taxon classificationAnimaliaAraneaeAraneidae

Mi & Wang sp. nov.

5298CA2D-284F-5B6B-8A12-C0ACED933869

https://zoobank.org/A6E7BBBE-E0D1-4053-8662-C710A05D25AD

Figs 1, 2, 3, 4, 5

##### Chinese name.

建新副楚蛛.

##### Type material.

***Holotype***: China • ♂; Xizang Auton. Region, Shigatse City, Gyirong Co., Gyirong Township, Nai Vill.; 28°24.43'N, 85°20.83'E, ca 3,310 m a.s.l.; 18 Aug. 2024; X.Q. Mi et al. leg.; TRU-Araneidae-411. ***Paratypes***: • 5♀♀8♂♂; same data as for holotype; TRU-Araneidae-412–424; 11♀17♂, Gyirong Township, Zhuo Vill.; 28°31.11'N, 85°13.05'E, ca 3,330 m a.s.l.; 4 Aug. 2019; X.Q. Mi & C. Wang leg.; TRU-Araneidae-425–452; • 1♀, Gyirong Township, Zhuo Vill.; 28°22.87'N, 85°19.60'E, ca 2,790 m a.s.l.; 17 Aug. 2024; X.Q. Mi et al. leg.; TRU-Araneidae-453; • 6♀♀8♂♂, Zongga Township, Xia Vill.; 28°34.91'N, 85°15.73'E, ca 3,800 m a.s.l.; 20 Aug. 2024; X.Q. Mi et al. leg.; TRU-Araneidae-454–467.

**Figure 1.**
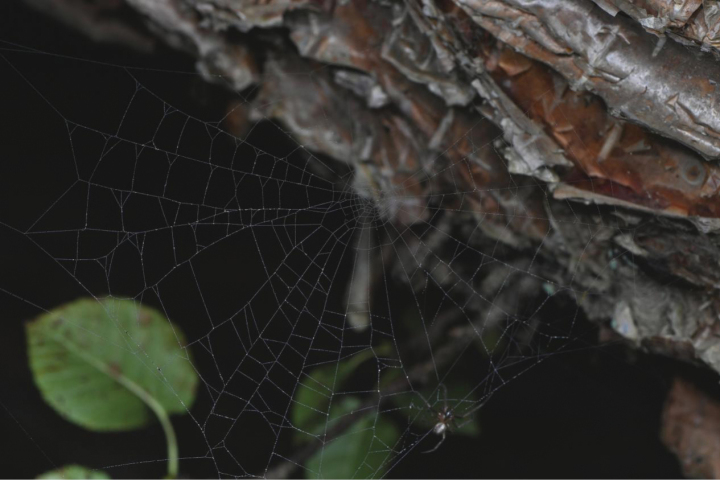
Web of *Parazygiella jianxini* Mi & Wang, sp. nov. Photo by X.Q. Mi.

**Figure 2.**
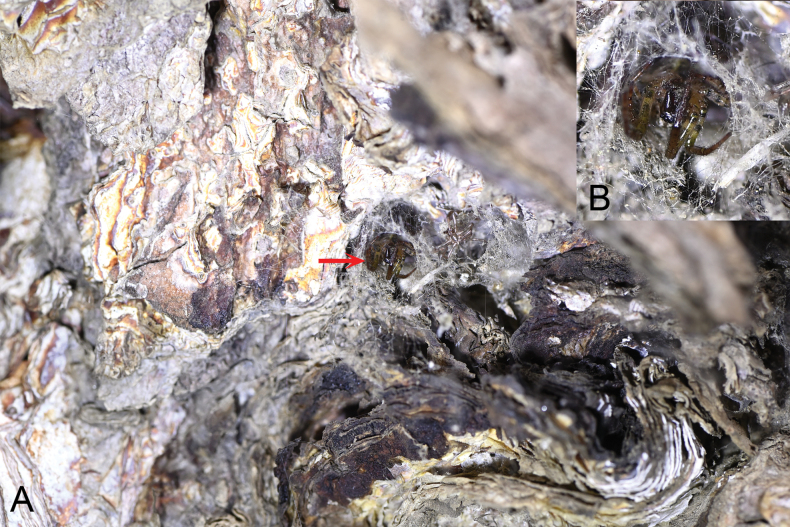
Retreat of *Parazygiella jianxini* Mi & Wang, sp. nov. **A**. Red arrow points to the spider; **B**. Spider in the retreat. Photo by X.Q. Mi.

**Figure 3.**
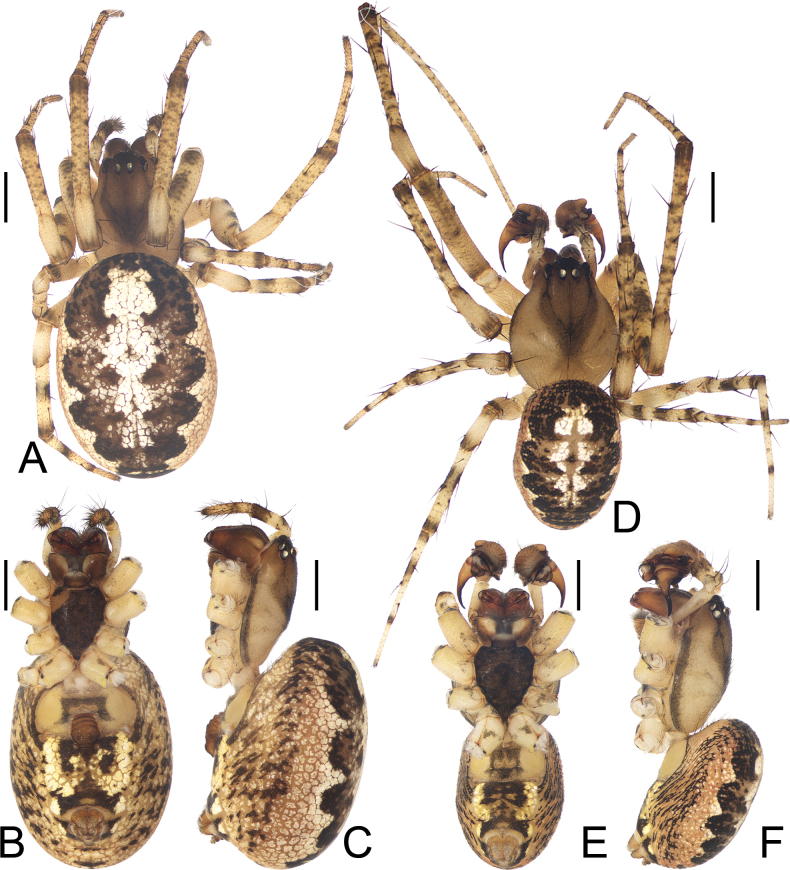
*Parazygiella jianxini* Mi & Wang, sp. nov. **A–C**. Female paratype TRU-Araneidae-412; **D–F**. Male holotype; **A, D**. Habitus, dorsal view; **B, E**. Ibid., ventral view; **C, F**. Ibid., lateral view. Scale bars: 1 mm.

##### Etymology.

The species is named after Professor Jianxin Li, a forestry expert (Tongren University, China).

##### Diagnosis.

**Figure 4.**
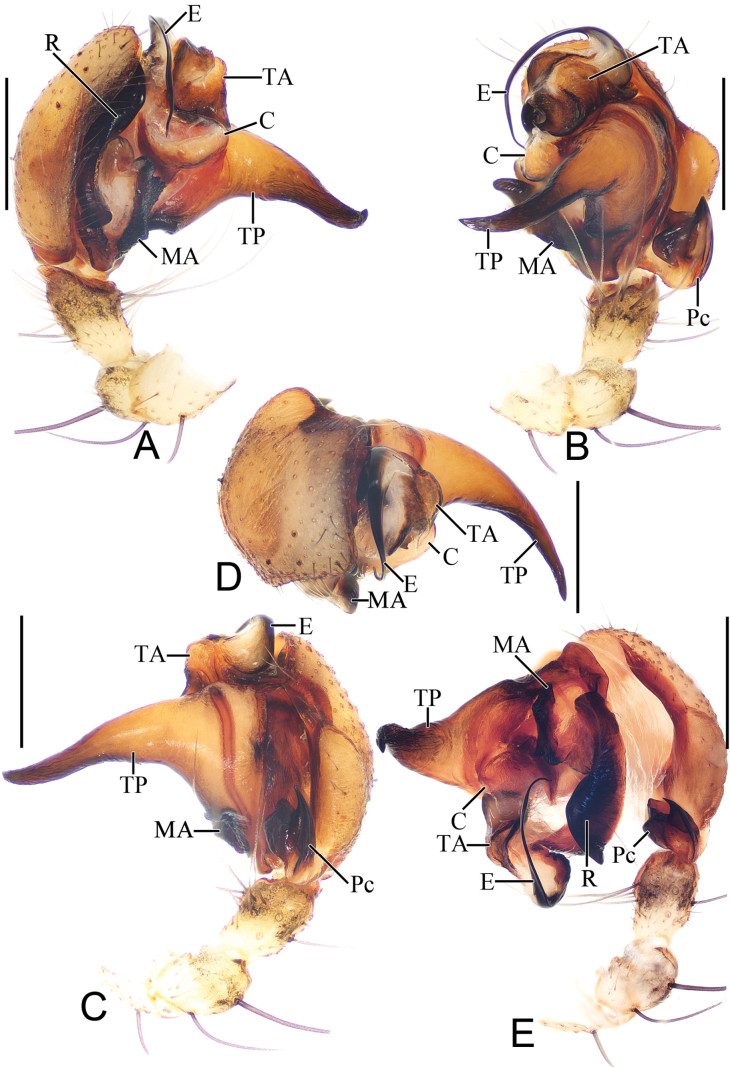
*Parazygiella jianxini* Mi & Wang, sp. nov. male holotype. **A**. Left palp, prolateral view; **B**. Ibid., ventral view; **C**. Ibid., retrolateral view; **D**. Ibid., apical view; **E**. Ibid., expanded. Abbreviations: C = conductor, E = embolus, MA = median apophysis, Pc = paracymbium, R = radix, TA = terminal apophysis, TP = tegular projection. Scale bars: 0.5 mm.

**Figure 5.**
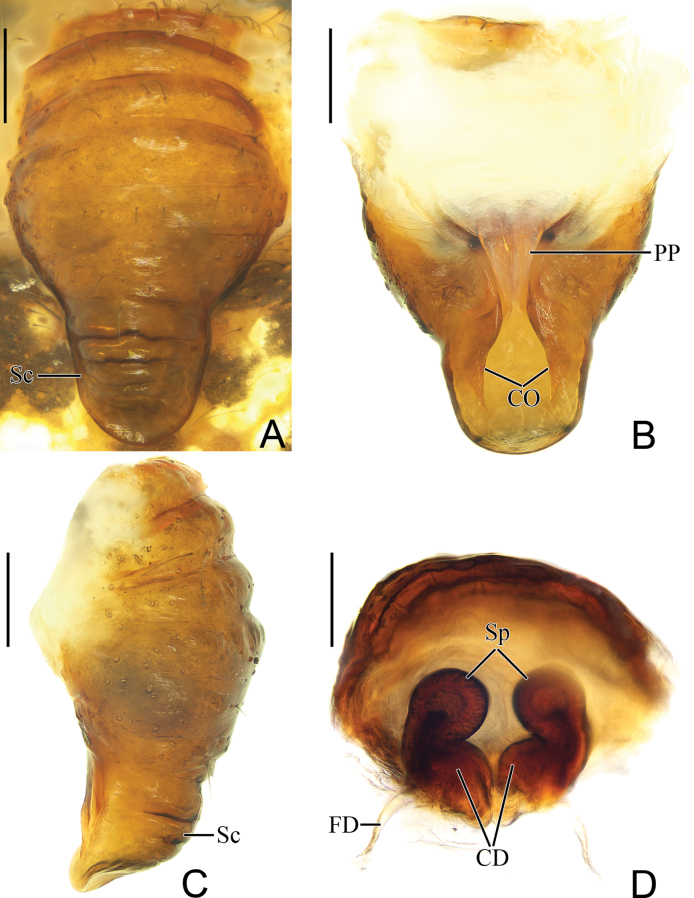
*Parazygiella jianxini* Mi & Wang, sp. nov. female paratype TRU-Araneidae-412. **A**. Epigyne, ventral view; **B**. Ibid., dorsal view; **C**. Ibid., lateral view; **D**. Vulva, anterodorsal view. Abbreviations: CD = copulatory duct, CO = copulatory opening, FD = fertilization duct, PP = posterior plate, Sc = scape, Sp = spermatheca. Scale bars: 0.1 mm.

*Parazygiella jianxini* Mi & Wang, sp. nov. resembles *P. yanghongae* Mi & Wang, sp. nov. in both somatic and morphology of copulatory organs, but it can be distinguished by having the tegular projection (TP) about 4/5 of the cymbial length (Fig. 4C) vs half of cymbial length (Fig. 7C); epigyne widest at middle part (Fig. 5A) vs widest at basal part (Fig. 8A); and posterior plate (PP) ~2.50 × wider at anterior than posterior (Fig. 5B) vs ~1.36 × wider at anterior than posterior (Fig. 8B).

##### Description.

**Male** (holotype, Figs 3D–F, 4A–E). Total length 5.50. Carapace 2.80 long, 2.10 wide. Abdomen 3.30 long, 2.10 wide. Clypeus 0.13 high. Eye sizes and interdistances: AME 0.20, ALE 0.13, PME 0.15, PLE 0.13, AME–AME 0.13, AME–ALE 0.10, PME–PME 0.10, PME–PLE 0.20, MOA length 0.48, anterior width 0.48, posterior width 0.40. Leg measurements: I 14.80 (3.70, 4.90, 4.40, 1.80), II 10.70 (2.90, 3.60, 2.90, 1.30), III 6.80 (2.10, 2.20, 1.60, 0.90), IV 8.40 (2.50, 2.90, 2.00, 1.00). Carapace greyish brown in cephalic region and greyish yellow in thoracic region (Fig. 3D, F). Cervical groove conspicuous (Fig. 3D). Chelicerae dark brown, with three teeth on both promargin and retromargin (Fig. 3E, F). Endites with tooth anterolaterally, brown at base, with paler tip; labium dark brown at base, with paler tip (Fig. 3E). Sternum dark brown (Fig. 3E). Legs yellow with grey annuli and spots (Fig. 3D). Abdomen ~1.57× longer than wide, dorsal patches extended from anterior to posterior, dark brown with white median longitudinal band (Fig. 3D, F). Ventral abdomen yellow to dark brown with paired white bracket-shaped marks laterally (Fig. 3E). Spinnerets yellowish (Fig. 3E, F).

***Palp*** (Fig. 4A–E): femur ~4.0× longer than wide (Fig. 3D–F); patella equal in length and width (Fig. 4A–C, E); tibia ~1.3× longer than wide in ventral view (Fig. 4A–C, E); cymbium ~1.26× longer than wide, retrolateral edge swollen (Fig. 4A–E); paracymbium heavily sclerotized, divided into two lobes with sharp distal edges (Fig. 4B, C, E); bulb ~1.56× longer than wide in ventral view; tegulum with long, horn-shaped projection (TP), about as long as 4/5 of cymbium length (Fig. 4A–E); radix (R) long and curved, heavily sclerotized, running parallel to edge of cymbium; median apophysis (MA) situated near base of bulb, short, rod-shaped, heavily sclerotized, with approximately 10 ridges distally (Fig. 4A, B, E); embolus long, flattened, with wide, translucent base, curved over tip of bulb and extending basally nearly half of bulb length (Fig. 4A–E); conductor (C) translucent, roughly square (Fig. 4A, B, D, E); terminal apophysis (TA) slightly wider than long in ventral view, arched angularly over tip of tegulum (Fig. 4A–E).

**Female** (paratype TRU-Araneidae-412, Figs 3A–C, 5A–D). Total length 6.80. Carapace 2.80 long, 2.00 wide. Abdomen 4.80 long, 3.30 wide. Clypeus 0.08 high. Eye sizes and interdistances: AME 0.18, ALE 0.13, PME 0.17, PLE 0.13, AME–AME 0.13, AME–ALE 0.10, PME–PME 0.10, PME–PLE 0.18, MOA length 0.50, anterior width 0.48, posterior width 0.43. Leg measurements: I 10.40 (2.90, 3.70, 2.60, 1.20), II 8.10 (2.40, 2.80, 1.90, 1.00), III 5.50 (1.80, 1.80, 1.10, 0.80), IV 6.90 (2.10, 2.50, 1.50, 0.80). Habitus similar to that of male (Fig. 3A–C).

***Epigyne*** (Fig. 5A–D): ~1.49× longer than wide in ventral view, slightly convex, widest at middle part, with transverse ridges and grooves; scape about 1/2 epigynal base width, round distally (Fig. 5A, C); posterior plate (PP) smooth, ~2.50× wider at anterior than posterior (Fig. 5B); copulatory openings slit-like, situated on dorsal surface of scape (Fig. 5B); copulatory ducts (CD) slightly curved, connected to spermathecae at anterodorsal surface (Fig. 5D); spermathecae rounded, about 1/3 diameters apart (Fig. 5D); fertilization ducts (FD) longer than diameter of spermatheca.

##### Variation.

Total length: ♂♂ 5.30–6.10 (*N* = 34), ♀♀ 6.80–8.00 (*N* = 23).

##### Natural history.

The specimens were mainly found on webs between trunks of large trees during the night; they hide in retreats under bark in the daytime or when disturbed. The webs have about 25 radii (Figs 1, 2).

##### Distribution.

China (Xizang) (Fig. 9).

#### Parazygiella yanghongae

Taxon classificationAnimaliaAraneaeAraneidae

Mi & Wang sp. nov.

3579287C-1073-55E7-B599-96B8D0841A69

https://zoobank.org/1CFB20FF-1F87-4CF4-98FC-C1AC2714155B

Figs 6, 7, 8

##### Chinese name.

杨红副楚蛛.

##### Type material.

***Holotype***: China • ♂; Xizang Auton. Region, Shigatse City, Yadong Co., Xiasima Township, Chunpi Vill.; 27°28.30'N, 88°54.53'E, ca 2,920 m a.s.l.; 9 Aug. 2019; X.Q. Mi & C. Wang leg.; TRU-Araneidae-468. ***Paratypes***: • 3♀♀2♂♂; same data as for holotype; TRU-Araneidae-469–473.

##### Etymology.

The specific name is the full name of Professor Hong Yang, a forestry expert (Tongren University, China).

##### Diagnosis.

*Parazygiella yanghongae* Mi & Wang, sp. nov. resembles *P. jianxini* Mi & Wang, sp. nov. in both somatic and morphology of copulatory organs, but it can be distinguished by having the tegular projection (TP) about 1/2 of the cymbial length (Fig. 7C) vs 4/5 of cymbial length (Fig. 4C); and epigyne widest at basal part (Fig. 8A) vs widest at middle part (Fig. 5A).

##### Description.

**Figure 6.**
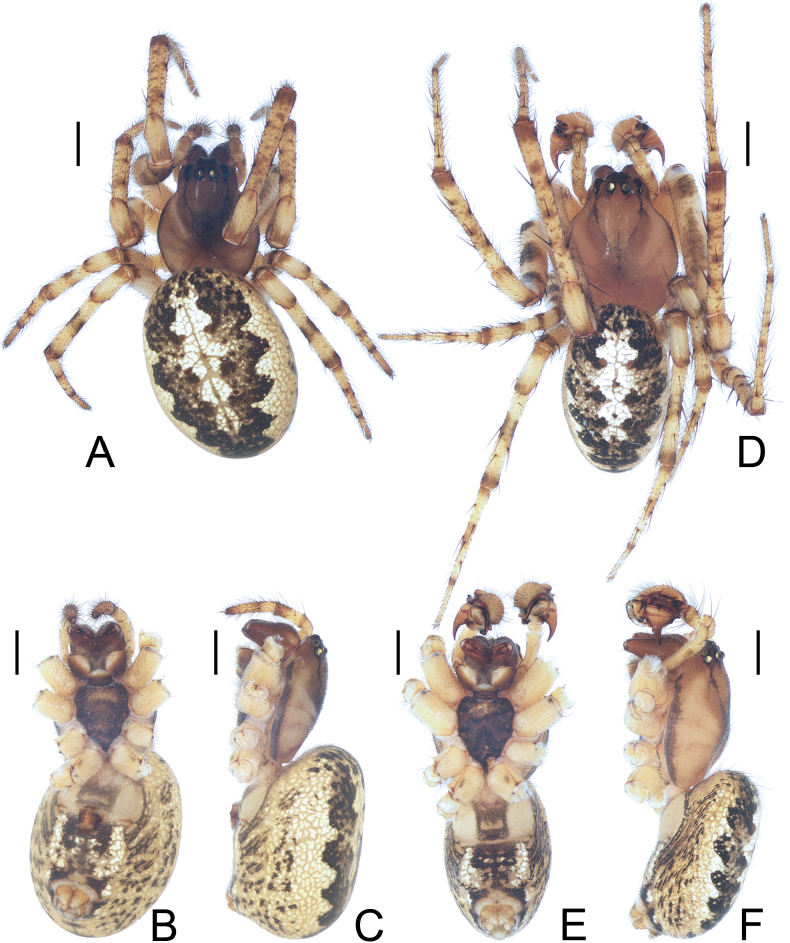
*Parazygiella yanghongae* Mi & Wang, sp. nov. **A–C**. Female paratype TRU-Araneidae-469; **D–F**. Male holotype; **A, D**. Habitus, dorsal view; **B, E**. Ibid., ventral view. **C, F**. Ibid., lateral view. Scale bars: 1 mm.

**Figure 7.**
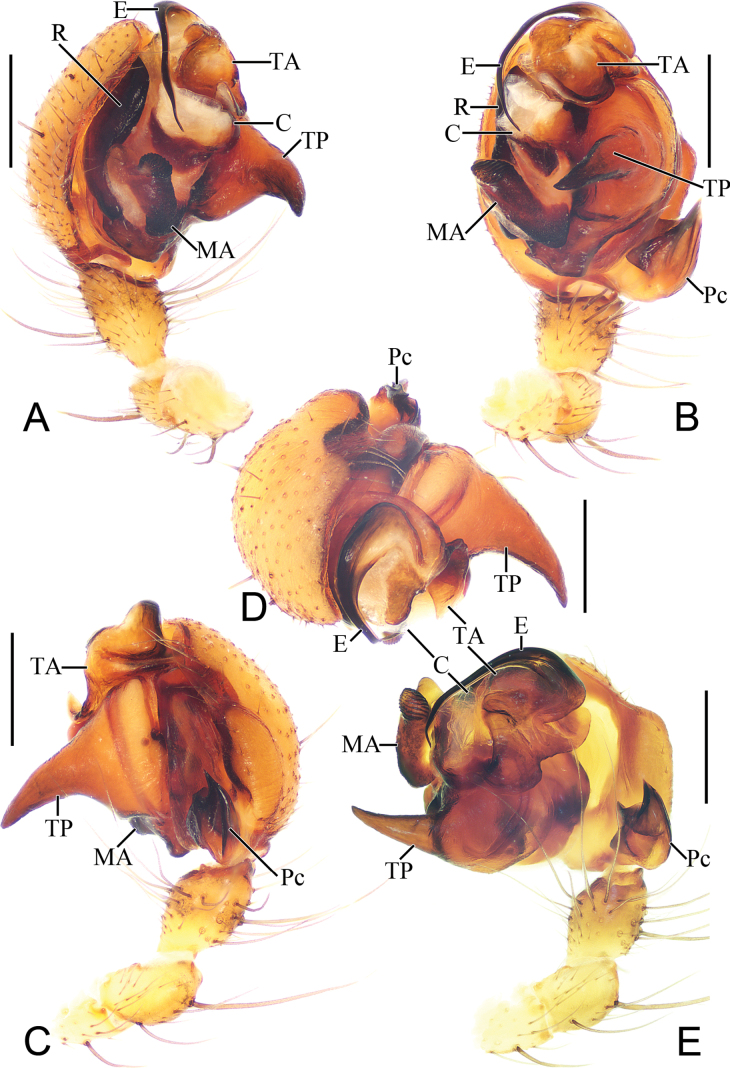
*Parazygiella yanghongae* Mi & Wang, sp. nov. male holotype. **A**. Left palp, prolateral view; **B**. Ibid., ventral view; **C**. Ibid., retrolateral view; **D**. Ibid., apical view; **E**. Ibid., expanded. Abbreviations: C = conductor, E = embolus, MA = median apophysis, Pc = paracymbium, R = radix, TA = terminal apophysis, TP = tegular projection. Scale bars: 0.5 mm.

**Male** (holotype, Figs 6D–F, 7A–E). Total length 6.80. Carapace 3.40 long, 2.60 wide. Abdomen 4.00 long, 2.40 wide. Clypeus 0.10 high. Eye sizes and interdistances: AME 0.23, ALE 0.15, PME 0.18, PLE 0.15, AME–AME 0.13, AME–ALE 0.08, PME–PME 0.15, PME–PLE 0.20, MOA length 0.53, anterior width 0.53, posterior width 0.50. Leg measurements: I 16.20 (4.20, 5.70, 4.70, 1.60), II 12.20 (3.30, 4.20, 3.40, 1.30), III 7.60 (2.40, 2.50, 1.70, 1.00), IV 9.40 (2.90, 3.30, 2.20, 1.00). Carapace yellowish brown, with darker cephalic region (Fig. 6D, F). Cervical groove conspicuous (Fig. 6D). Chelicerae dark brown, with three promarginal and two retromarginal teeth (Fig. 6E, F). Endites with tooth anterolaterally, brown at base, with paler tip; labium dark brown at base, with paler tip too (Fig. 6E, F). Sternum dark brown (Fig. 6E). Legs yellow with brown annuli and spots (Fig. 6D). Abdomen ~1.32× longer than wide, dorsal patches extended from anterior to posterior, dark brown with white median longitudinal band (Fig. 6D, F). Venter of abdomen yellow to dark brown with paired white bracket-shaped marks laterally (Fig. 6E). Spinnerets yellowish (Fig. 6E, F).

***Palp*** (Fig. 7A–E): femur ~4.0× longer than wide (Fig. 6D–F); patella as long as wide (Fig. 7A–C, E); tibia as wide as long in ventral view (Fig. 7A–C, E); cymbium ~1.1× longer than wide, retrolateral edge swollen (Fig. 7A–E); paracymbium heavily sclerotized, divided into two lobes with sharp distal edges (Fig. 7B–E); bulb ~1.3× longer than wide in ventral view; tegulum with long, horn shaped projection (TP), about as long as 1/2 cymbium length (Fig. 7A–E); radix (R) long and curved, heavily sclerotized, running parallel to edge of cymbium; median apophysis (MA) situated near base of bulb, short, rod-shaped, heavily sclerotized, with dozen of ridges distally (Fig. 7A, B, E); embolus long, flattened, with wide, translucent base, curved over tip of tegulum and extending basally nearly half of bulb length (Fig. 7A, B, D, E); conductor (C) translucent, roughly square (Fig. 7A, B, D, E); terminal apophysis (TA) about 3/5 of bulb width in ventral view, arched angularly over tip of bulb (Fig. 7A–E).

**Figure 8.**
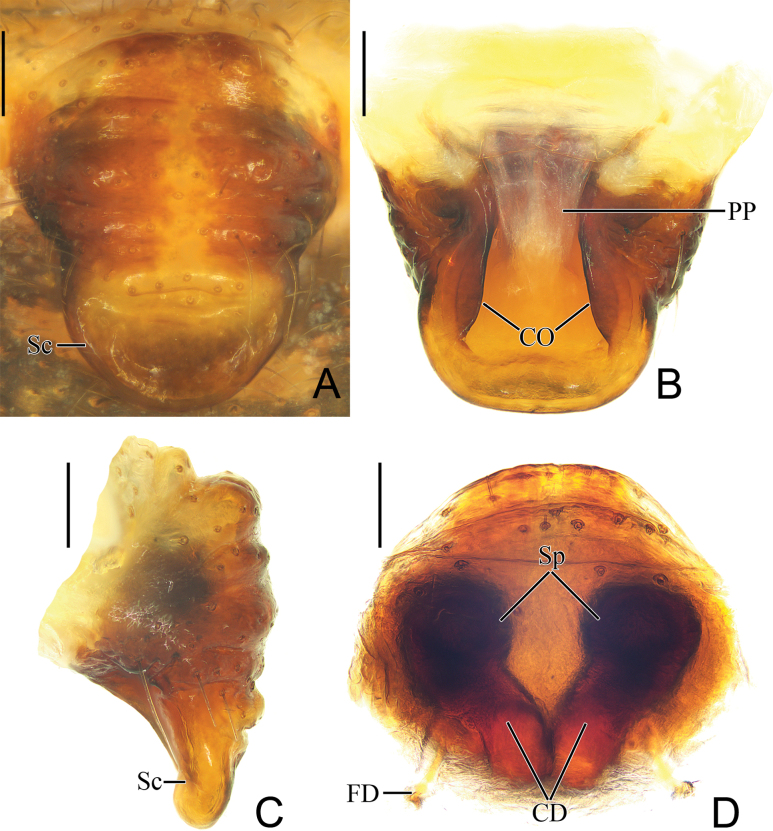
*Parazygiella yanghongae* Mi & Wang, sp. nov. female paratype TRU-Araneidae-469. **A**. Epigyne, ventral view; **B**. Ibid., dorsal view; **C**. Ibid., lateral view; **D**. Vulva, anterodorsal view. Abbreviations: CD = copulatory duct, CO = copulatory opening, FD = fertilization duct, PP = posterior plate, Sc = scape, Sp = spermatheca. Scale bars: 0.1 mm.

**Female** (paratype TRU-Araneidae-469, Figs 6A–C, 8A–D). Total length 6.90. Carapace 3.00 long, 2.30 wide. Abdomen 4.50 long, 3.40 wide. Clypeus 0.10 high. Eye sizes and interdistances: AME 0.23, ALE 0.15, PME 0.18, PLE 0.15, AME–AME 0.08, AME–ALE 0.08, PME–PME 0.13, PME–PLE 0.18, MOA length 0.50, anterior width 0.48, posterior width 0.48. Leg measurements: I 10.80 (3.00, 3.90, 2.70, 1.20), II 8.70 (2.50, 3.00, 2.20, 1.00), III 5.80 (1.90, 1.90, 1.20, 0.80), IV 7.70 (2.50, 2.70, 1.70, 0.80). Habitus similar to that of male (Fig. 6A–C).

***Epigyne*** (Fig. 8A–D): ~1.15× longer than wide in ventral view, slightly convex, widest at basal part, with few transverse ridges and grooves, scape about 7/10 epigynal base width, round distally (Fig. 8A, C); posterior plate smooth, ~1.36× wider at anterior than posterior (Fig. 8B); copulatory openings slit-like, situated on dorsal surface of scape (Fig. 8D); copulatory ducts slightly curved, connected to spermathecae at anterodorsal surface (Fig. 8D); spermathecae rounded, about 1 radius apart (Fig. 8D); fertilization ducts (FD) about equal length to diameter of spermatheca.

##### Variation.

Total length: ♂♂ 6.50–6.80 (*N* = 3), ♀♀ 6.20–6.90 (*N* = 3).

##### Natural history.

The spiders weave webs on tall roadside trees during the night.

##### Distribution.

**Figure 9.**
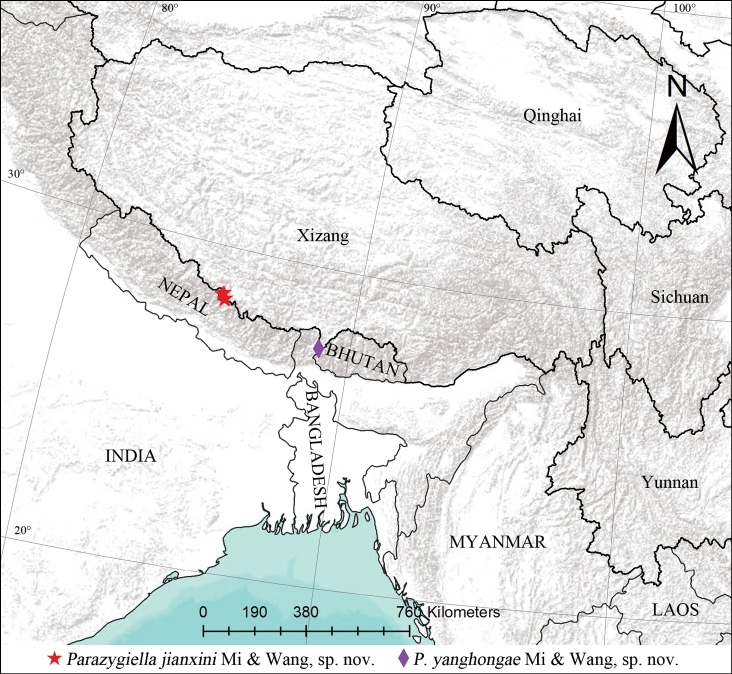
Distribution in China of the new species of *Parazygiella*.

China (Xizang) (Fig. 9).

##### Note.

Based on the close similarities in both somatic and morphology of copulatory organs of *Zygiella hiramatsui* Tanikawa, 2017 and *Z. nearctica* Gertsch, 1964 to the type species of *Parazygiella*, *P. montana*, such as longitudinal dorsal patches on elliptical abdomen, paracymbium divided into two lobes distally, presence of terminal apophysis (Tanikawa 2017), together with the molecular phylogenetic evidence (Kallal and Hormiga 2018; Kallal et al. 2020), we propose the following new combinations: *P. hiramatsui* (Tanikawa, 2017), comb. nov. and *P. nearctica* (Gertsch, 1964), comb. nov.

#### Parazygiella hiramatsui

Taxon classificationAnimaliaAraneaeAraneidae

(Tanikawa, 2017) comb. nov.

AD6A861A-689E-5FB9-8D57-61C8C6BA7E92

Zygiella hiramatsui Tanikawa, 2017 (Basionim).

#### Parazygiella nearctica

Taxon classificationAnimaliaAraneaeAraneidae

(Gertsch, 1964) comb. nov.

049C9D5D-B115-546D-86B6-FDF7BB9CC4DE

Zygiella nearctica Gertsch, 1964 (Basionim).

## Supplementary Material

XML Treatment for Parazygiella

XML Treatment for Parazygiella jianxini

XML Treatment for Parazygiella yanghongae

XML Treatment for Parazygiella hiramatsui

XML Treatment for Parazygiella nearctica

## Additional information

### Use of AI

No use of AI was reported.

### Author ORCIDs

Xiaoqi Mi https://orcid.org/0000-0003-1744-3855

Cheng Wang https://orcid.org/0000-0003-1831-0579

### Data availability

All of the data that support the findings of this study are available in the main text.
